# High-Intensity Aerobic Exercise Suppresses Cancer Growth by Regulating Skeletal Muscle-Derived Oncogenes and Tumor Suppressors

**DOI:** 10.3389/fmolb.2022.818470

**Published:** 2022-06-21

**Authors:** Hyunseok Jee, Eunmi Park, Kyunghoon Hur, Minjeong Kang, Yoosik Kim

**Affiliations:** ^1^ School of Kinesiology, Yeungnam University, Gyeongsan, South Korea; ^2^ College of Life Science and Nano Technology, Hannam University, Daejeon, South Korea; ^3^ Department of Chemical and Biomolecular Engineering, Korea Advanced Institute of Science and Technology (KAIST), Daejeon, South Korea; ^4^ KAIST Institute for Health Science and Technology (KIHST), KAIST, Daejeon, South Korea

**Keywords:** exercise, cancer, RNA-seq, skeletal muscle, tumor suppressors

## Abstract

High-intensity aerobic exercise (90% of the maximal heart rate) can effectively suppress cancer cell proliferation *in vivo*. However, the molecular effects of exercise and its relevance to cancer prevention remain uninvestigated. In this study, mice with colorectal cancer were subjected to high-intensity aerobic exercise, and mRNA-seq analysis was performed on the heart, lungs, and skeletal muscle tissues to analyze the genome-wide molecular effects of exercise. The skeletal muscle-derived genes with exercise-dependent differential expression were further evaluated for their effects on colorectal cancer cell viability. Compared to the results obtained for the control groups (healthy and cancer with no exercise), the regular and high-intensity aerobic physical activity in the mice produced positive results in comprehensive parameters (i.e., food intake, weight gain, and survival rate). A heatmap of differentially expressed genes revealed markedly different gene expression patterns among the groups. RNA-seq analysis of 23,282 genes expressed in the skeletal muscle yielded several anticancer effector genes (e.g., *Trim63*, *Fos*, *Col1a1*, and *Six2*). Knockdown and overexpression of selected anticancer genes repressed CT26 murine colorectal carcinoma cell proliferation by 20% (*p* < 0.05). Our findings, based on the aerobic exercise cancer mouse model, suggest that high-intensity aerobic exercise results in a comprehensive change in the expression patterns of genes, particularly those that can affect cancer cell viability. Such an approach may identify key exercise-regulated genes that can help the body combat cancer.

## 1 Introduction

Exercise is a positive effector of physical fitness, which contributes to improved health. The effects of exercise involve muscular functions that affect the overall well-being of the body, including mental and emotional health (Solberg et al., 2013; Izquierdo et al., 2020). However, exercise can also have adverse effects on health, depending on exposure to exercise stimulation. A customized exercise regime can be established by selecting optimized conditions, such as time, intensity, frequency, and style of exercise. This principle can be applied in cancer prevention and treatment. Cancer-related outcomes such as anxiety, depression, fatigue, and quality of life can also be improved by specific evidence-based exercise modalities by optimizing FITT (frequency, intensity, time, type) (Campbell et al., 2019). Different modes of exercise can be ideal for different types of cancer. Previous studies have shown that proper exercise can positively serve individuals with cancer (Newton and Galvao, 2008; Jee and Kim, 2019; Schmitz et al., 2019). For example, a recent microarray-based study on the effect of low-intensity resistance training on human subjects showed that the accurate mode and extent of exercise resulted in the upregulation of microRNAs, such as miR-630 and miR-5703, and myokines (fractalkine/CX3CL1) among 42 pathways and 12 cytokines/myokines, which subsequently suppressed tumor growth by inducing tumor-specific cytotoxic T cells (Hashida et al., 2021). In addition, the ladder climbing resistant training exercise on preclinical tumor-bearing animals also mitigated muscle atrophy, tumor growth, and cancer malignancy (Padilha et al., 2019; Padilha et al., 2021). However, the molecular mechanisms underlying the anticancer effects of exercise remain largely unknown.

The muscle is one of the most important organs required for physical performance (Park et al., 2012). Muscle tissues are classified as slow or fast types based on their myosin heavy chain isoforms, and the ratio of these two muscle types affects athletic performance. The different ratios of the muscle types may be related to the heterogeneous expression of specific genes (Cohen et al., 2015). The gastrocnemius muscle, considered in this study, is a fast-type skeletal muscle and consists of a balanced ratio of fast (78%∼95%) and slow (5%∼22%) fibers (Jee et al., 2009; Jee et al., 2016b). Such muscle is chosen to offset the possibility of having one-sided muscle fibers, which may influence gene expression. By secreting compounds such as myokines, metabolites, and exosomes, the muscle tissue can function as an endocrine organ and affects the entire body in response to exercise (Raschke et al., 2013; Hartwig et al., 2014). Currently, it is unknown how the gene expression pattern of skeletal muscles is affected by high-intensity aerobic exercise and how such changes may help the body combat cancer.

Every day, cells in the human body experience mutations that may induce jeopardizing and uncontrolled growth. This is the basic notion of cancer. Cancers can develop in various tissues such as the oral cavity, digestive, respiratory, reproductive, derma, blood, and more. In addition, different kinds of cancer have unique etiology, such as human papillomavirus causing cervical cancer (Yang and Jee, 2021). Carcinogenesis in the muscle, however, is extremely rare even though sarcomas do exist. In fact, there is no cancer in the cardiac muscle; it is the colonized cells in the myocyte that develop into cancer (Willis, 1952). Therefore, we hypothesized that muscle-specific factors could suppress cancer cell attachment and that muscle contraction-driven exercise may affect this phenomenon.

Several studies have shown the benefits of exercise, particularly in enhancing the immune system to prevent cancer. Exercise affects body composition, endocrine secretion, systematic inflammation, and immune cell function (Sitlinger et al., 2020). In particular, exercise can lead to an influx of immune cells that result in a 60% reduction in tumor incidence and growth (Pedersen et al., 2016). Acute aerobic exercise rapidly increases immune cell counts in blood and results in interleukin-6 dependent redistribution of NK cells, ultimately suppressing the tumor growth (Karre et al., 1986; Walsh et al., 2011; Pedersen et al., 2016). However, only a limited number of studies have explored the molecular effects of exercise (Koelwyn et al., 2015; Idorn and Thor Straten, 2017). These studies focused on the epigenetic effects of aerobic exercise and the potential effects on the expression of specific downstream genes such as interleukins (ILs), tumor necrosis factor-alpha (TNF-α), interferon-gamma (IFNγ), and transforming growth factor. Other studies evaluated the effect of aerobic and resistance exercise on the expression of skeletal muscle genes using mRNA-seq (Popov et al., 2018; Pillon et al., 2020). However, they failed to identify the key genes that could mediate the anticancer effects of exercise. This is the key rationale of our study that we combined the aerobic exercise and cancer mouse models to unravel the anticancer genes regulated by aerobic exercise in a genome-wide manner.

In this study, we used mRNA-seq to profile gene expression changes following high-intensity aerobic exercise in mice. In particular, we analyzed the gastrocnemius muscle, heart, and lungs to identify genes potentially mediating the anticancer effects of exercise. By comparing the effects of exercise in normal and colorectal cancer-bearing mice, we analyzed the genetic changes stimulated by exercise and the presence of cancer. Furthermore, we identified genes that are regulated both by exercise and cancer, but opposite in direction. By modulating the expression of these genes *in vitro*, we analyzed their potential anticancer effects. These findings provide evidence of colorectal cancer-related gene expression changes *in vivo* and suggest potential molecular effectors of exercise that may have anticancer activity in this colorectal cancer animal model.

## 2 Materials and Methods

### 2.1 Animals and Exercise Protocol

Forty pathogen-free male CDF1 mice, weighing 18–20 g, at 4 weeks of age were purchased from the Central Animal Laboratory (Seoul, Korea). The mice were randomly divided into no-exercise non-cancer healthy control group [N = 10; E(−)T(−)], no exercise with cancer group [N = 10; E(−)T(+)], high-intensity aerobic exercise healthy group [N = 10; E(+) T(−)], and high-intensity aerobic exercise with cancer group [N = 10; E(+)T(+)]. All animals were bred in our pathogen-free animal facilities and allowed access to standard chow and water *ad libitum*. They were housed in a sterile room maintained at 22–24°C with a 12:12 h light-dark cycle. For the treadmill exercise, a modification of a published protocol was used (Zogaib and Monte-Alto-Costa, 2011). The mice were made to run on a four-lane-motorized treadmill with a light shield (45 min with 0° slope, 09:00∼12:00 p.m.) once every 2 days in order to minimize the effect of biorhythms and overwork. High-intensity aerobic exercise was conducted at 1.0 km/h (90% of the maximal heart rate). Animals were sacrificed at the end of the experiments, on day 19, by exsanguination under anesthesia induced by a mixture of tiletamine, zolazepam, and xylazine (40 mg/kg body mass). Tissues, including the right gastrocnemius muscles, lungs, and hearts of each group, were isolated for further analyses.

Animal use and maintenance protocols were approved by the Yeungnam University Medical Centre Institutional Animal Care and Use Committee (YUMC-AEC2020-008). The evaluation criteria were as follows: rationale and purpose of the proposed animal use; justification of species and number of animals requested; unnecessary duplication of tests or experiments; availability or appropriateness of the use of minimally invasive procedures; adequacy of training and personnel experience; multiple major surgical procedures conducted; unusual housing and husbandry requirements; appropriate sedation, analgesia, and anesthesia; method of euthanasia or disposition of animals; timely intervention criteria and procedures or euthanasia, if required; and safety of the working environment for personnel.

### 2.2 Study Design

The gastrocnemius muscles, lungs, and hearts of the four different groups of mice were analyzed through mRNA-seq. In total, 23,282 genes were identified. Of these, 40 genes (*Col3a1*, *Col5a2*, *IL15*, *Tnnc1*, *Col1a1*, *Osr2*, *Ifrd1*, *Trim63*, *Asb2*, *Maff*, *Myl6b*, *Six2*, *Fos*, *etc.*) were selected based on at least two-fold changes and normalized read count of four in all three-way comparisons 1) the effect of high-intensity aerobic exercise [E(+)T(−) vs. E(−)T(−)], 2) cancer effect [E(−)T(+) vs. E(−)T(−)], and 3) the effect of exercise on cancer-bearing mice [E(+)T(+) vs. E(−)T(+)] groups in the gastrocnemius muscle. The expression of these genes was further verified *via* quantitative reverse transcription-polymerase chain reaction (RT-qPCR) analysis of samples from littermates of the original four groups. The selected genes were used for the Kyoto Encyclopedia of Genes and Genomes (KEGG) mapper (https://www.genome.jp/kegg/mapper.html). Based on the mRNA-seq, RT-qPCR validation, and KEGG analysis data, four genes (*Fos*, *Trim63*, *Six2*, and *Col1a1*) were examined for their potential effects on cancer cell viability.

### 2.3 Establishing the Mouse Cancer Model

Approximately 1 × 10^5^ CT26 murine colorectal cancer cells (purchased from Korean Cell Bank, Seoul, Korea) were injected into each mouse *via* the tail vein to establish the cancer mouse model. The mice were then returned to their cages. They were periodically examined with the VISQUE InVivo Smart imaging system (Vieworks, Chayon, Korea), following the injection of MMPSense680 reagent (PerkinElmer, Waltham, MA, United States) *via* the tail vein to examine cancer cell metastasis.

### 2.4 Tissue-to-Body Weight Ratio, Body Weight Change, and Food Intake

Bodyweight and food intake were monitored to assess the quality of life. The lung, heart, and gastrocnemius muscles were isolated at the end of the study. The weights of these tissues were determined, and the weight differences among the groups were compared (Table 1).

**TABLE 1:** Tissue weight data.

		Normal control	Cancer control	Cancer exercise	Normal exercise
Spleen		75.50 ± 3.69	181.00 ± 19.86	184.00 ± 30.82*	78.33 ± 5.75
Diaphragm		70.33 ± 4.03	269.25 ± 122.65	568.00 ± 130.19	65.29 ± 5.83
Plantaris muscle	R	11.67 ± 0.99	10.00 ± 0.69	13.17 ± 0.83	15.29 ± 0.68 Ω ^∞∞^
	L	12.83 ± 0.60	8.28 ± 0.52^##^	11.00 ± 1.02^∇∇^	15.42 ± 0.43^∞∞^
Gastrocnemius muscle	R	113.17 ± 5.96	85.29 ± 3.76^##^	88.71 ± 6.09*^∇∇^	127.17 ± 3.75^∞∞^
	L	111.33 ± 4.92	82.43 ± 4.59^##^	93.00 ± 5.54^∇∇^	128.33 ± 3.77^∞∞^

N = 4–10; Values are mean ± S.D. Note that Diaphragm muscle showed remarkably increased weight in the cancer groups even though there are no significant differences. Only the right of right hindlimb plantaris muscle shows the statistical difference between control and exercise; *p < 0.05: statistical significance between control and cancer exercise; ^Ω^ *p* < 0.05: statistical significance between control and exercise; ^##^ *p* < 0.01: statistical significance between control and cancer; ^∞∞^ *p* < 0.01: statistical significance between exercise and cancer; ^∇∇^ *p* < 0.01: statistical significance between exercise and cancer exercise.

### 2.5 RNA Extraction

Total RNA from each sample was extracted using TRIzol reagent (Thermo Fisher Scientific, Waltham, MA, United States) according to the manufacturer’s instructions. The quality of the extracted RNA was assessed using a model 2100 bioanalyzer equipped with an RNA 6000 Nano Chip (Agilent Technologies, Santa Clara, CA, United States). RNA quantification was performed using an ND-2000 spectrophotometer (Thermo Fisher Scientific, Waltham, MA, United States).

### 2.6 Library Preparation and Sequencing

The sequencing library was constructed using the QuantiSeq 3′ mRNA-Seq Library Prep Kit (Lexogen Inc., Vienna, Austria) according to the manufacturer’s instructions. Total RNA (500 ng) was extracted, and an Illumina platform-compatible sequence containing an oligo-dT primer was hybridized for reverse transcription. An Illumina-compatible linker sequence containing random sequences was used as the posterior procedure for degradation of the RNA template. Magnetic beads were used to purify the double-stranded sequencing library. Complete adapter sequences for cluster generation were added to amplify the library. The high-throughput sequencing procedure (as the single-end 75 sequences) was then performed using the NextSeq 500 device (Illumina Inc., San Diego, CA, United States). mRNA-seq analysis identified 23,282 genes. Changes in their expression levels were calculated as log_2_ values for genes with higher than normalized read count of four.

### 2.7 Cell Culture and Transfection

CT26 cells were grown in Dulbecco’s modified Eagle’s medium (DMEM), supplemented with 10% fetal bovine serum, 100 IU/ml penicillin, and 100 mg/ml streptomycin in a 5% CO_2_/95% air humidified atmosphere. Cells were passaged every 3–4 days and maintained at a dilution of 1.5 × 10^5^ cells per well (90 mm in diameter) via trypsinization until the cells reached approximately 70%–80% confluency. For transfection, Lipofectamine 3000 (Thermo Fisher Scientific, Waltham, MA, United States) was used according to the manufacturer’s instructions. The sequences of the small interfering RNAs (siRNAs) used in this study are provided in Supplementary Table S1. For overexpression of a target gene, pcDNA3 plasmid vectors encoding the complementary DNA of the gene were transfected into the cells using Lipofectamine 3000. Prior to every experiment, the cells were cultured for at least 18 h for stabilization.

### 2.8 RT-qPCR

Approximately 1 μg of total RNA was reverse transcribed with RevertAid reverse transcriptase (Thermo Fisher Scientific, Waltham, MA, United States) to synthesize cDNA. RT-qPCR was performed using SYBR Green to analyze changes in gene expression. The primer sequences used in this study are listed in Supplementary Table S2.

### 2.9 Cell Viability Assay

For adherent cell lines, 4.0 × 10^4^ cells were seeded per well in a 24-well plate and stabilized for 18 h. 3-(4,5-dimethylthiazol-2-yl)-2,5-diphenyltetrazolium bromide) (MTT) assay (CyQUANT™ MTT Cell Viability Assay, Thermo Fisher Scientific, Waltham, MA, United States) was performed, following the manufacturer’s instructions, after transfection and incubation of siRNA and cDNA plasmids for 72 h. Cell viability was determined by measuring the absorbance at 580 nm using a Varioskan LUX multimode microplate reader (Thermo Fisher Scientific, Waltham, MA, United States).

### 2.10 Western Blotting

CT26 cell lysates were prepared by suspending the cell pellets in lysis buffer (50 mM Tris-HCl, pH 8.0, 100 mM KCl, 0.5% NP-40, 10% glycerol, and 1 mM DTT), supplemented with a protease inhibitor cocktail (Merck, Darmstadt, Germany), and incubating on ice for 10 min. Complete lysis was achieved by sonication. Protein samples (40 µg) were separated *via* sodium dodecyl sulfate-polyacrylamide gel electrophoresis and transferred onto a poly(vinylidene fluoride) membrane (Merck, Darmstadt, Germany) using an Amersham semi-dry transfer system. The primary antibodies used in this study were as follows: Fos (Cell Signaling Technology, Danvers, MA, United States, 4384T), Col1a1 (Cell Signaling Technology, Danvers, MA, United States, 91144S), Six2 (LSBio, Seattle, WA, United States, LS-C386068), Trim63 (R&D Systems, Minneapolis, MN, United States, AF5366), and β-tubulin (Cell Signalling Technology, Danvers, MA, United States, 86298S).

### 2.11 Data and Statistical Analyses

Bowtie2 sequence alignment was performed using QuantSeq 3′ mRNA-Seq reads (Langmead and Salzberg, 2012). Genome assembly sequence or the representative transcript sequence derived from the Bowtie2 indices was generated to map the sequencing reads to the genome. The aligned files for assembling transcripts estimated their abundance and detected the differential expression of genes. Differentially expressed genes (DEGs) were determined using coverage in Bedtools derived counts (Quinlan and Hall, 2010). Using Bioconductor (Gentleman et al., 2004), read count data were processed according to the quantile normalization method using EdgeR within R (Law et al., 2016). DAVID (http://david.abcc.ncifcrf.gov/) was used for gene ontology analysis of DEGs.

All data are presented as mean ± standard error of the mean. Two-way repeated measure ANOVA was used to calculate the *p*-values for the daily food intake and weight of the mice. *p*-values for the tissue weight data in Table 1 were determined *via* a one-way ANOVA with a one-sided Dunnett’s test by comparing the experimental groups to the normal control group. A student’s t-test was used to determine *p*-values for the data presented in Figure 6.

## 3 Results

### 3.1 High-Intensity Aerobic Exercise Prevents Cancer Development *In Vivo*

**FIGURE 1:**
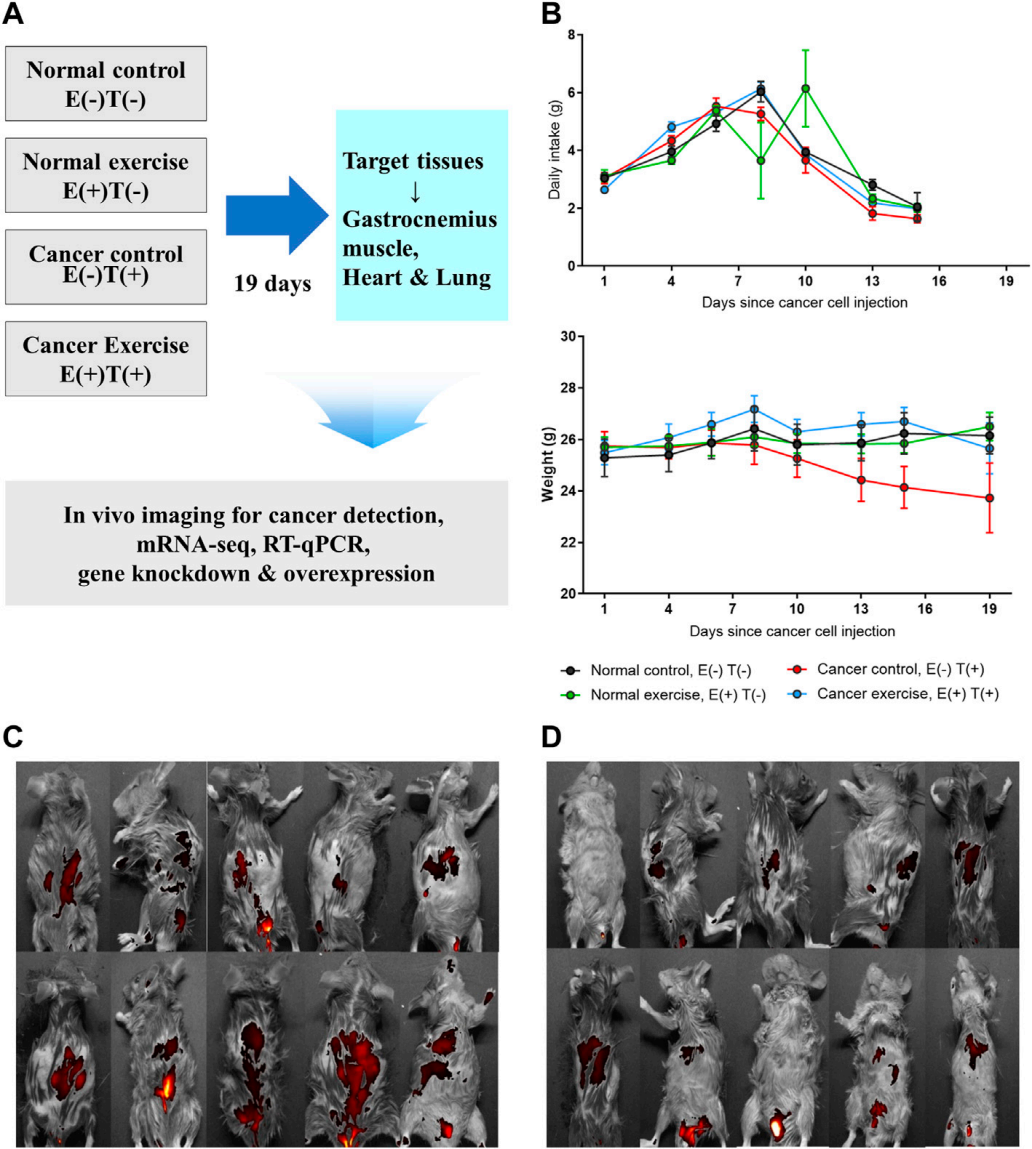
*In vivo* animal model to study the molecular effect of high-intensity aerobic exercise. **(A)** In the current study, 40 mice were divided into four groups of ten mice each (normal control, normal exercise, cancer control, and cancer exercise). The changes of daily intake and weight of each group were traced for 19 days. After 19 days, *in vivo* images for detecting CT-26-derived cancer metastasis were taken for cancer-bearing mice, and tissues were isolated for detecting proteins, mRNA-seq, RT-qPCR, *in vitro* gene downregulation and/or overexpression analyses. **(B)** Average daily food intake and weight changes of mice from each group during the 19 days of the experiment. Statistical differences (*p* = 0.007 for food intake and *p* = 0.001 for weight changes) in the interaction between the number of days and the group are shown. **(C,D)** *In vivo* images showing that cancer metastasized the whole mice in cancer control **(C)** and cancer exercise mice used in this study **(D)**.

We applied increasing physical fitness to imitate regular physical activity in a cancer mouse model to examine the molecular effects of exercise on cancer development. To induce cancer, we introduced CT26 murine colorectal cancer cells in mice *via* tail vein injection, which resulted in the dissemination of cancer throughout the body, including the colon and lungs. Throughout the study, we compared four groups of mice (Figure 1A: 1) healthy mice without exercise [Normal control; E(−)T(−)], 2) cancer-bearing mice without exercise [Cancer control; E(−)T(+)], 3) cancer-bearing mice with high-intensity aerobic exercise [Cancer exercise; E(+)T(+)], and 4) healthy mice with high-intensity aerobic exercise [Normal exercise; E(+)T(−)]. High-intensity aerobic exercise was performed on a motorized treadmill with a maximal heart rate of 90%, which is a standard load for the high-intensity aerobic exercise based on the estimated maximum oxygen consumption (Zogaib and Monte-Alto-Costa, 2011). We monitored the changes in food intake and weight to analyze the effects of cancer and high-intensity aerobic exercise (Figure 1B). Significant differences were observed in body weights and food intake among the four groups during the exercise intervention (*p* < 0.01). Mice in the cancer control group [E(−)T(+)] showed a dramatic weight loss over the course of the experiment. In contrast, mice in the high-intensity aerobic exercise group maintained their weight regardless of cancer development. In addition, we observed significantly different interactions with food intake among the four groups over time (*p* < 0.01) (Figure 1B). Metastasis was confirmed in cancer-induced mice; however, the degree of tumor growth was significantly reduced in the high-intensity aerobic exercise group (Figure 1C). The images of all cancer-bearing mice, with or without high-intensity aerobic exercise, are shown in Figures 1C,D.

To further analyze the molecular effects of high-intensity aerobic exercise, specifically to identify the cancer-preventive effectors of exercise, we sacrificed the mice at the end of the experiment (19 days after the vein injection) and examined tissues obtained from the spleen, diaphragm, plantaris, and gastrocnemius muscle (Figure 1A; Table 1). The weight of spleen tissue in the cancer exercise group was approximately 2.44 times higher than that in the control group (*p* < 0.05). Examination of the distal part of the right and left skeletal muscles (plantaris and gastrocnemius muscles) revealed that hypertrophy was induced to a greater extent in the high-intensity aerobic exercise group than in the no-exercise group (*p* < 0.01). This result indicated cancer-induced atrophy in the gastrocnemius muscle rather than in the plantaris muscle. While both legs showed a significant reduction of the gastrocnemius muscle, only the plantaris muscle of the left leg decreased significantly (*p* < 0.01). Exercise did not influence hypertrophy in the cancer groups [E(−)T(+) vs. E(+)T(+)]. However, hypertrophy of the diaphragm in the cancer control mice was approximately 3.84 times more than that in the healthy control mice (Table 1). Hypertrophy of the diaphragm in cancer with high-intensity aerobic exercise groups was about eight times greater than that in the healthy control group (Table 1).

### 3.2 Changes in Gene Expression Patterns Induced by High-Intensity Aerobic Exercise

**FIGURE 2:**
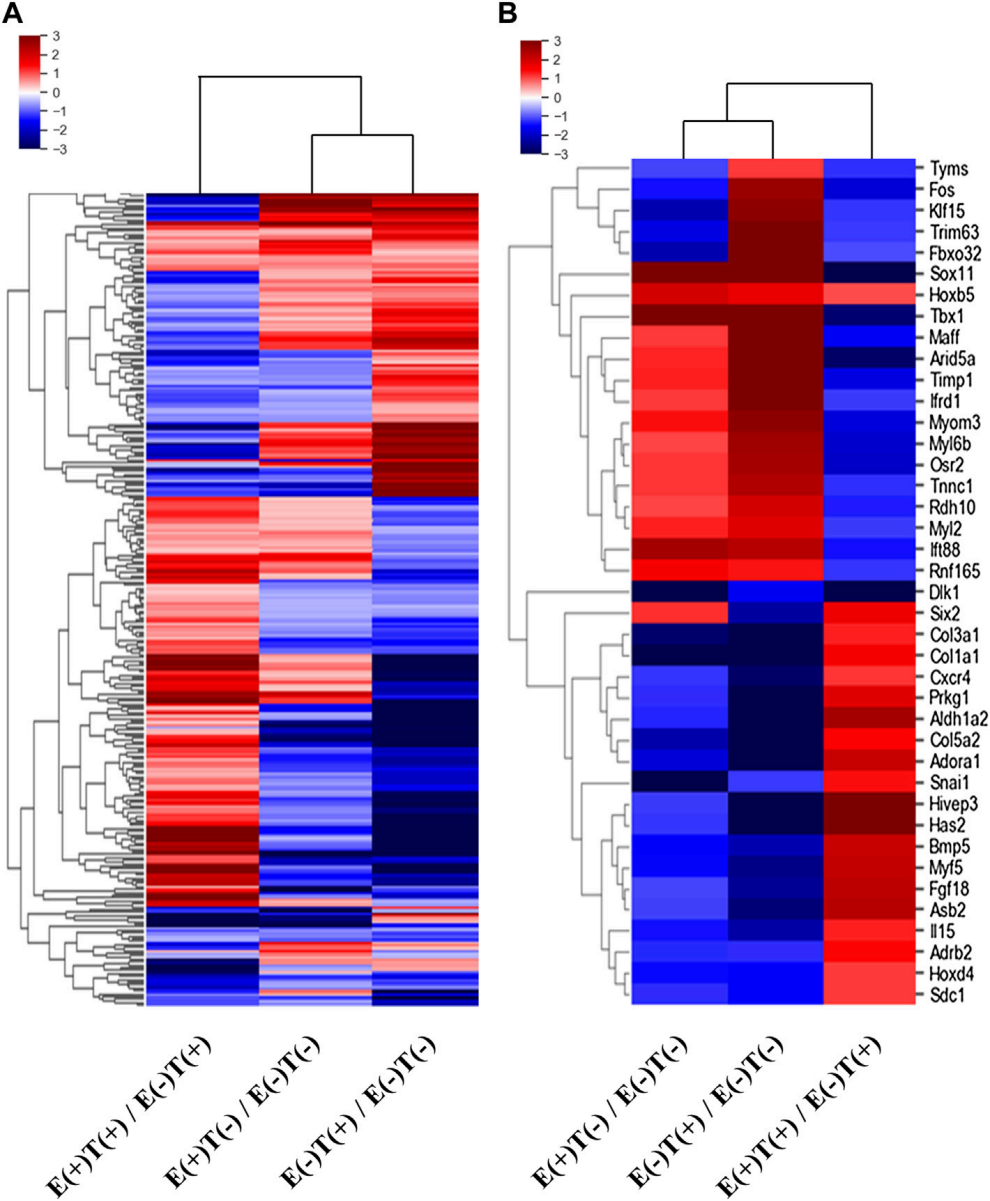
Heatmaps of DEGs of skeletal muscle-related genes. The differentially expressed skeletal muscle-related genes were selected according to the fold changes and normalized read counts from the output of mRNA-seq of skeletal muscles (gastrocnemius muscle). E, exercise; T, tumor injection; (+), execution; (−), non-execution. **(A)** A heatmap of top 285 DEGs (at least 1.25-fold change in expression in any one of the three-way comparisons shown) from 998 skeletal muscle-related genes. **(B)** A heatmap of top 40 DEGs (at least two-fold change in expression in all three-way comparisons) from 998 skeletal muscle-related genes. Remarkably different expressional patterns of the gastrocnemius muscle-derived genes are shown.

We isolated the gastrocnemius muscles from each group and extracted total RNA after homogenization. We then constructed mRNA-seq libraries using oligo-dT beads and analyzed the gene expression patterns *via* high-throughput sequencing. Each sample was analyzed using ExDEGA, including gene ontology (GO), which defines skeletal muscle-related genes from a pool of 23,282 genes. Skeletal muscle-related genes were identified using the QuickGO annotation list, which yielded 16 skeletal muscle-related terms defining a total of 336,468 annotations (https://www.ebi.ac.uk/QuickGO/). This analytical method yielded the expression patterns of 998 skeletal muscle-related genes. Clustering analysis was performed on these 998 genes, and a heatmap was constructed (Figure 2). The left column describes the expression pattern of the top 285 from the 998 genes selected with a minimum normalized read count of four and 1.25 fold change against that of the control group (Figure 2A). The red indicates upregulated genes, and the blue indicates downregulated genes. Similarly, the list was narrowed down by selecting genes that showed at least 2-fold changes in all three-way pairwise comparisons (Figure 2B).

### 3.3 Gene Expression Patterns in Tissues From the Exercise Intervention Mouse Cancer Model

**FIGURE 3:**
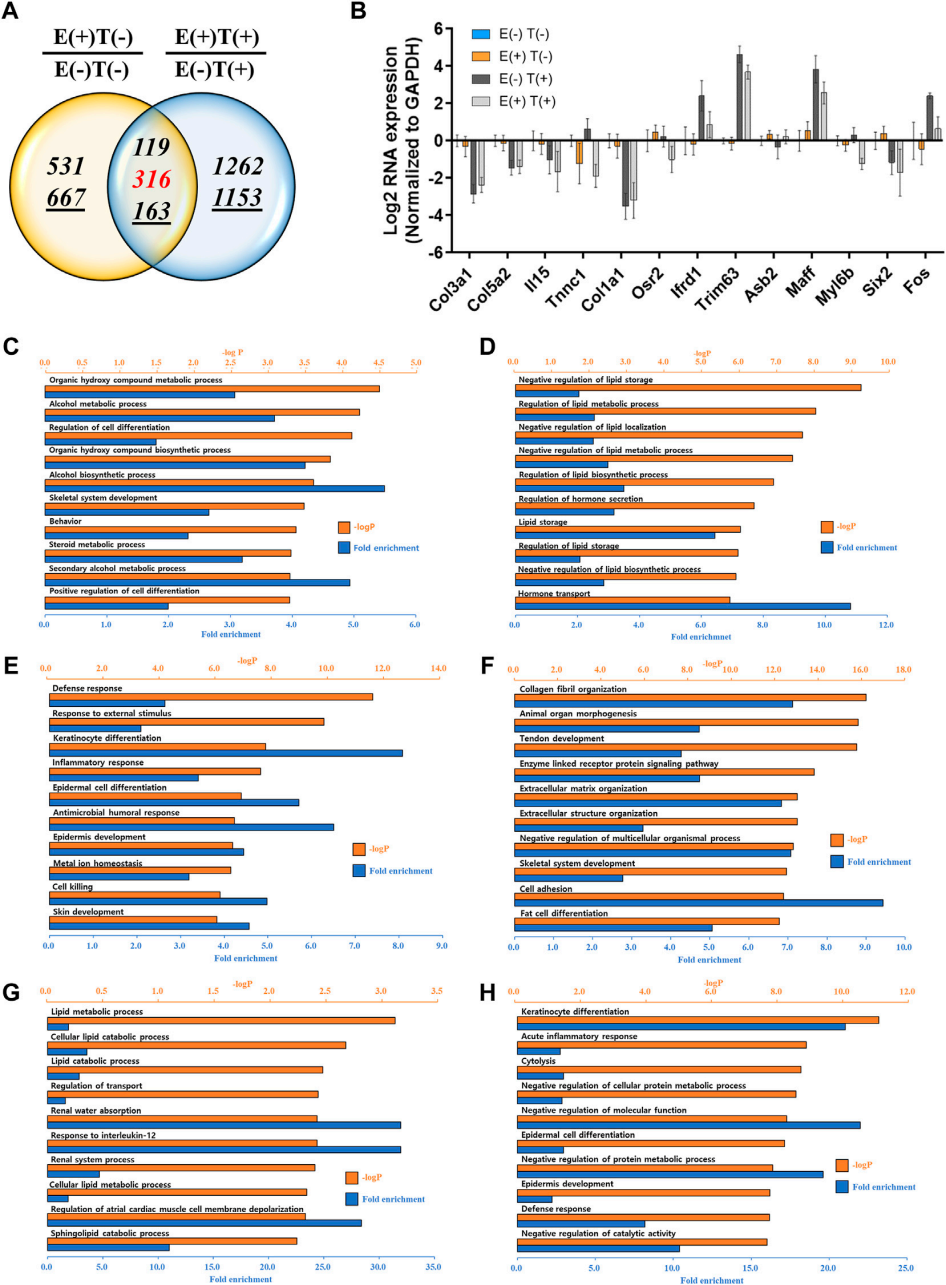
mRNA-seq analysis of skeletal (gastrocnemius) muscle tissue. **(A)** Venn diagram of the number of DEGs for the effect of exercise for normal [E(+)T(−)/E(−)T(−)] and tumor-bearing [E(+)T(+)/E(−)T(+)] mice. The high-intensity aerobic exercise resulted in 1,796 up- or downregulated genes by at least two-fold, while tumor-bearing mice resulted in a change in the 3,013 genetic expressions by at least two-fold. The italicized number indicates the number of upregulated genes while the italicized and underlined number indicates the number of downregulated genes. Those with opposite effects between the two groups (contra-regulated) are shown in red. The DEGs were defined as genes with a fold change of two and a normalized read count of four. **(B)** Bar graph of log2 fold change of selected genes in the contra-regulated group using RT-qPCR of the littermates. The fold change was calculated by normalizing the data to that of the housekeeping gene (glyceraldehyde 3-phosphate dehydrogenase*, GAPDH*) and then to the level in the normal control group. A consistent change compared to the mRNA-seq analysis was observed. **(C,D)** GO analysis of the upregulated **(C)** or downregulated DEGs **(D)** comparing E(+)T(−) and E(−)T(−) mice. The significant enriched terms include biosynthetic hormone secretion and lipid-related processes. **(E,F)** GO analysis of the upregulated **(E)** or downregulated DEGs **(F)** comparing E(−)T(+) and E(−)T(−) mice. This result indicates that the presence of cancer results in the systemic modulation of immune function. **(G,H)** GO analysis of the upregulated **(G)** or downregulated DEGs **(H)** comparing E(+)T(+) and E(−)T(+) mice. Enrichment of immune-related terms is clear, suggesting that exercise may successfully prevent the global gene expression changes induced by cancer, particularly those related to immune function. For all GO analyses, the top 300 DEGs were used, and the top ten pathways ranked by –logP and fold enrichment are shown.

The gastrocnemius muscle was obtained from a representative mouse from each of the four groups, and the gene expression profiles were analyzed *via* mRNA-seq. We first analyzed the effect of high-intensity aerobic exercise on gene expression [E(+)T(−) vs. E(−)T(−)] and the effect of exercise on gene expression in cancer-bearing mice [E(+)T(+) vs. E(−)T(+)]. We found that high-intensity aerobic exercise resulted in up- or downregulation of 1,796 genes by at least two-fold, while in the cancer-bearing mice, exercise resulted in a change in the expression of 3,013 genes by at least two-fold (Figure 3A). Considering that no tumor growth was observed in the gastrocnemius muscle tissue of the cancer exercise mouse, bearing cancer results in a remarkable change in gene expression throughout the body.

Among these DEGs, we focused on the genes that showed a negative correlation between high-intensity aerobic exercise and the presence of tumors (shown in red in Figure 3A). These genes showed differential expression by more than two-fold when the mice underwent high-intensity aerobic exercise, but showed a reverse change when cancer was induced. In other words, these genes may represent the molecular factors under which exercise resulted in a cancer-preventive effect by reversing the changes induced by the presence of cancer. A few examples of genes with up- or downregulation and a negative correlation between exercise and cancer are shown in Figure 3B. We further validated the expression patterns of these genes by analyzing their expression in littermates using RT-qPCR, which showed a consistent change as in our mRNA-seq analysis (Figure 3B).

Next, we performed GO analysis on the DEGs in each pair. First, we compared gene expression in the healthy control mice and high-intensity aerobic exercise mice (Figures 3C,D). The significantly enriched terms for upregulated genes were related to the biosynthetic process and hormone transport, indicating that high-intensity aerobic exercise may induce systemic effects by increasing biosynthetic hormone secretion from the skeletal muscle (Figure 3C). In addition, the lipid-related process term was downregulated in mice undergoing high-intensity aerobic exercise (Figure 3D). Next, we compared the effect of cancer by comparing gene expression in healthy controls with that in cancer-bearing mice (Figures 3E,F). Significantly enriched terms included defense response, cell killing, and skin-related responses such as keratinocyte differentiation and skin development. This result indicates that the presence of cancer results in the systemic modulation of immune function. Lastly, when we compared gene expression in the cancer control mice with that in cancer and high-intensity aerobic exercise mice, the observed enrichment of immune-related terms are shown in the downregulated genes, suggesting that exercise may successfully prevent the global gene expression changes induced by cancer, particularly those related to immune function (Figures 3G,H).

**FIGURE 4:**
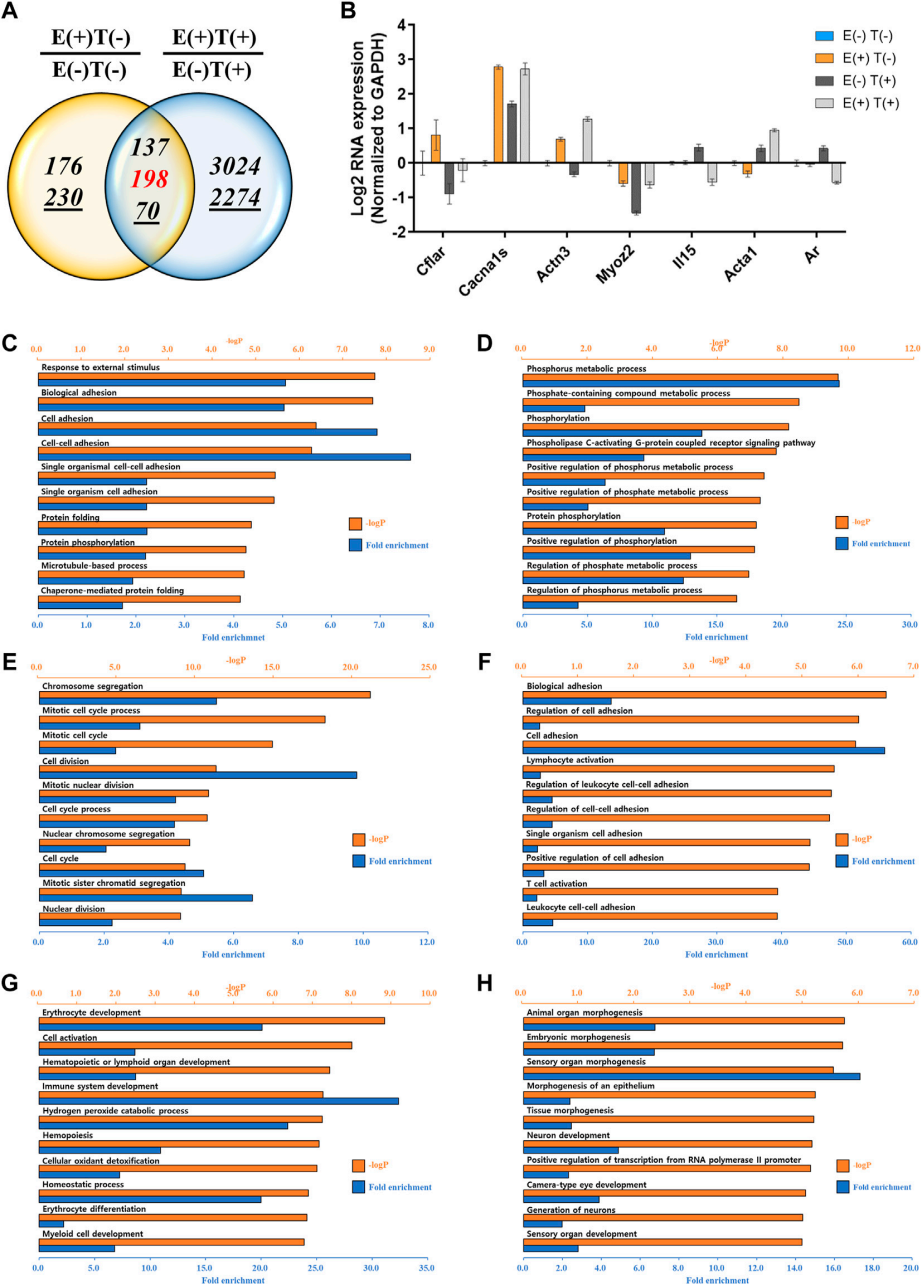
mRNA-seq analysis of the lungs. **(A)** Venn diagram of the number of DEGs for the effect of exercise for normal [E(+)T(−)/E(−)T(−)] and tumor-bearing [E(+)T(+)/E(−)T(+)] mice. The italicized number indicates the number of upregulated genes while the italicized and underlined number indicates the number of downregulated genes. Those with opposite effects between the two groups (contra-regulated) are shown in red. The DEGs were defined as genes with a fold change of two and a normalized read count of four. **(B)** Bar graph of log2 fold change of selected genes in the contra-regulated group using RT-qPCR of the littermates. The fold change was calculated by normalizing the data to that of *GAPDH* and then to the level in the normal control group. A consistent change compared to the mRNA-seq analysis was observed. **(C,D)** GO analysis of the upregulated **(C)** or downregulated DEGs **(D)** comparing E(+)T(−) and E(−)T(−) mice. Significantly enriched terms include cell adhesion, protein folding, and phosphorus metabolic process. **(E,F)** GO analysis of the upregulated **(E)** or downregulated DEGs **(F)** comparing E(−)T(+) and E(−)T(−) mice. The presence of cancer resulted in the upregulation of genes related to cell division while genes related to cell adhesion and T cell activation were downregulated. **(G,H)** GO analysis of the upregulated **(G)** or downregulated DEGs **(H)** comparing E(+)T(+) and E(−)T(+) mice. Exercise in cancer-bearing mice resulted in the upregulation of genes related to blood cell differentiation and cell activation while decreased genes associated with organ development. For all GO analyses, the top 300 DEGs were used, and the top ten pathways ranked by –logP and fold enrichment are shown.

**FIGURE 5:**
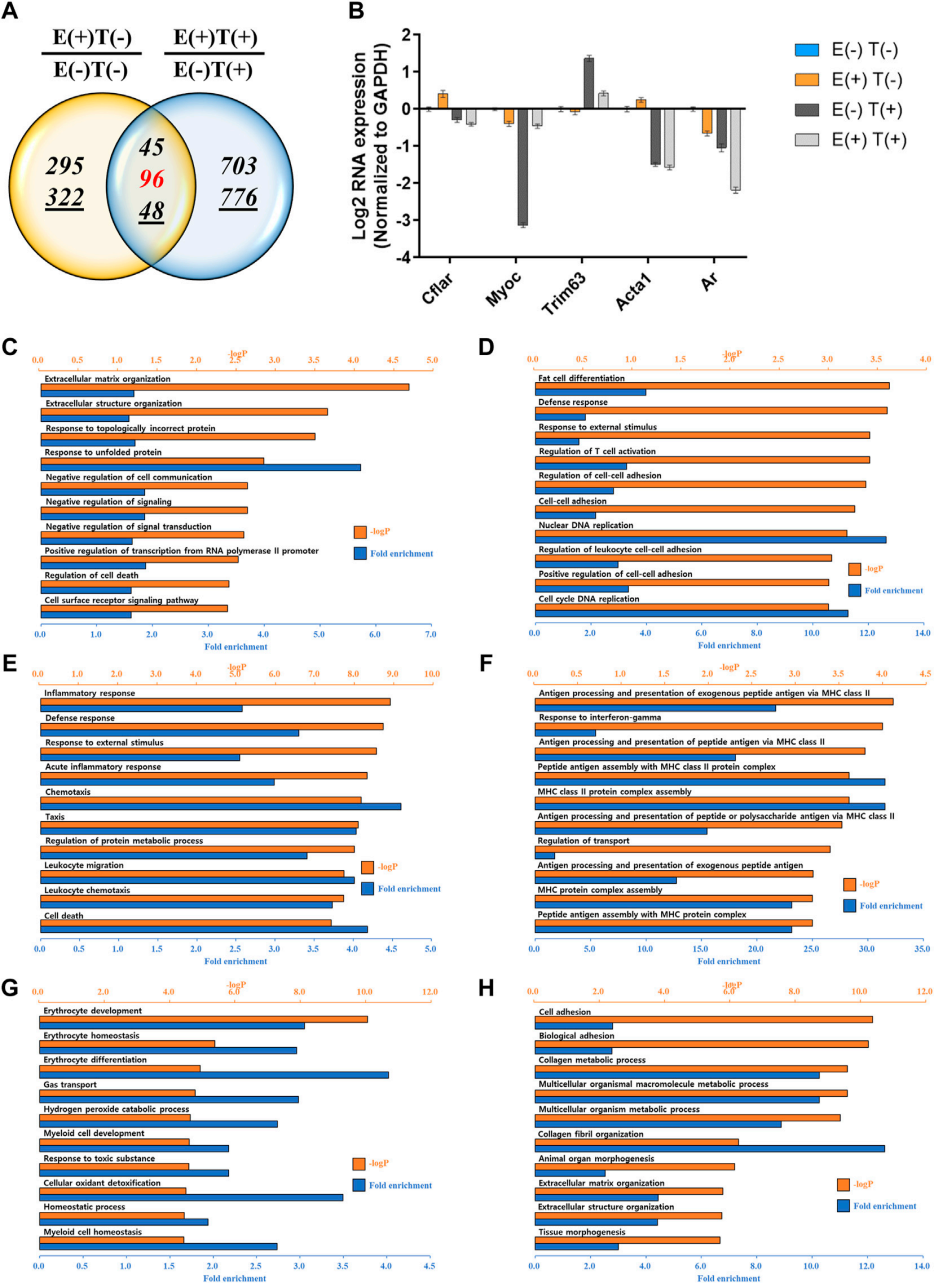
mRNA-seq analysis of the heart. **(A)** Venn diagram of the number of DEGs for the effect of exercise for normal [E(+)T(−)/E(−)T(−)] and tumor-bearing [E(+)T(+)/E(−)T(+)] mice. The italicized number indicates the number of upregulated genes while the italicized and underlined number indicates the number of downregulated genes. Those with opposite effects between the two groups (contra-regulated) are shown in red. The DEGs were defined as genes with a fold change of two and a normalized read count of four. **(B)** Bar graph of log2 fold change of selected genes in the contra-regulated group using RT-qPCR of the littermates. The fold change was calculated by normalizing the data to that of *GAPDH* and then to the level in the normal control group. A consistent change compared to the mRNA-seq analysis was observed. **(C,D)** GO analysis of the upregulated **(C)** or downregulated DEGs **(D)** comparing E(+)T(−) and E(−)T(−) mice. Exercise triggered the expression of genes related to protein folding and matrix reorganization while decreased genes associated with DNA replication and cell-cell adhesion. **(E,F)** GO analysis of the upregulated **(E)** or downregulated DEGs **(F)** comparing E(−)T(+) and E(−)T(−) mice. The presence of cancer resulted in inflammatory response and chemotaxis while decreased genes related to transport. **(G,H)** GO analysis of the upregulated **(G)** or downregulated DEGs **(H)** comparing E(+)T(+) and E(−)T(+) mice. Exercise in the cancer-bearing mice upregulated genes involved in detoxification and blood cell differentiation and downregulated genes involved in tissue morphogenesis and fibril organization. For all GO analyses, the top 300 DEGs were used, and the top ten pathways ranked by –logP and fold enrichment are shown.

We performed a similar analysis by examining gene expression profiles in other organs, such as the lungs and heart (Figures 4, 5). For the lungs, 810 up or downregulated genes were detected by filtering genes with a fold change of two and a normalized read count of four, owing to the high-intensity aerobic exercise effect, while 5,702 genes showed altered expression due to the effect of exercise in the cancer model (Figure 4A). Under the same conditions as in the lungs, the expression of 806 genes in E(+)T(−) vs. E(−)T(−) and 1,668 genes in E(+)T(+) vs. E(−)T(+) was altered in the heart tissue (Figure 5A). The RT-qPCR validation using the littermates for a selected DEGs (*Cflar*, *Cacna1s*, *Actn3*, *Myoz2*, *Il15*, *Acta1*, and *Ar* for the lungs; *Cflar*, *Myoc*, *Trim63*, *Acta1*, and *Ar* for the heart) from red-labeled contra-regulated genes (198 for the lungs and 96 for the heart) are shown in each figure (Figures 4B, 5B). Some genes from the heart and lungs were commonly regulated (i.e., *Cflar*, *Ar*, and *Acta1*).

GO analysis was also performed in the lungs and heart tissues as done for the gastrocnemius muscle. In the lungs, significantly enriched terms for upregulated genes by exercise were associated with cell adhesion and protein folding while downregulated genes by exercise were associated with the phosphorus metabolic process (Figures 4C,D). The presence of cancer resulted in the upregulation of genes related to cell division while genes related to cell adhesion and T cell activation were downregulated (Figures 4E,F). Notably, exercise in cancer-bearing mice resulted in the upregulation of genes related to blood cell differentiation and cell activation while decreased genes associated with organ development (Figures 4G,H). In the heart, exercise triggered the expression of genes related to protein folding and matrix reorganization while decreased genes associated with DNA replication and cell-cell adhesion (Figures 5C,D). At the same time, the presence of cancer resulted in inflammatory response and chemotaxis while decreased genes related to transport (Figures 5E,F). Lastly, exercise in the cancer-bearing mice upregulated genes involved in detoxification and blood cell differentiation and downregulated genes involved in tissue morphogenesis and fibril organization (Figures 5G,H). Based on the GO terms analyzed above, the observed systematic responses were differently enriched according to the cancer state, type of tissue, and exercise execution.

While analyzing our data, we noticed that *IL15* was commonly present in the contra-regulated group for gastrocnemius muscle and lungs, C*flar*, *Acta1*, and *Ar* were commonly present for lungs and heart, while *Trim63* was commonly present between gastrocnemius muscle and heart. In addition, among the DEGs, we found that *Rdh11*, *1700048E18Rik*, *Car3*, *Hcar1*, *Nup98*, and *Timp1* were commonly present in all three tissues.

### 3.4 Modulating the Expression of Exercise-Regulated Genes Affects Cancer Cell Viability

Further, we performed functional annotation analysis of the 18 RT-qPCR validated DEGs using KEGG Mapper annotations (*Col3a1*, *Col5a2*, *Ifrd1*, *Fos*, *Asb2*, *Tnnc1*, *Osr2*, *Maff*, *Six2*, *Trim63*, *Il15*, *Cflar*, *MyoC*, *Acta1*, *Ar*, *Cacna1s*, *Actn3*, and *Myoz2*). Their functions were then identified using the *Mus musculus* database. Overrepresented pathways involved ten genes: *Cflar*, *Fos*, *Actn3*, *Ar*, *Cacna1s*, *Col1a1*, *Col3a1*, *Col5a2*, *Il15*, and *Tnnc1*. Among these genes, all, except *Actn3* and *Tnnc3*, were relevant in signaling pathways related to cancer and the immune system, such as the nuclear factor-kappa B, tumor necrosis factor, mitogen-activated protein kinase (MAPK), Toll-like receptor, B- and T-cell receptor signaling, and PI3K-AKT signaling pathways (Supplementary Data S1).

We also performed KEGG pathway analysis on 285 skeletal muscle-related DEGs. Out of these 285 genes, 34 of them participate in major pathways in cancer, and 26 are involved in the metabolic pathways. When we focused on the differential expression induced by exercise [E(−)T(−) vs. E(+)T(−)], we found that many of the genes involved in the MAPK pathway are downregulated (Supplementary Data S2). Moreover, increased p53 which activates the apoptotic pathway, is also evident (Supplementary Data S2). Analysis using the STRING database (http://string-db.org/) revealed annotated interactions between 40 selected DEGs with other genes at the protein level (Supplementary Data S3). However, these connections were not evident in any of the pathways obtained in the KEGG Mapper annotations.

**FIGURE 6:**
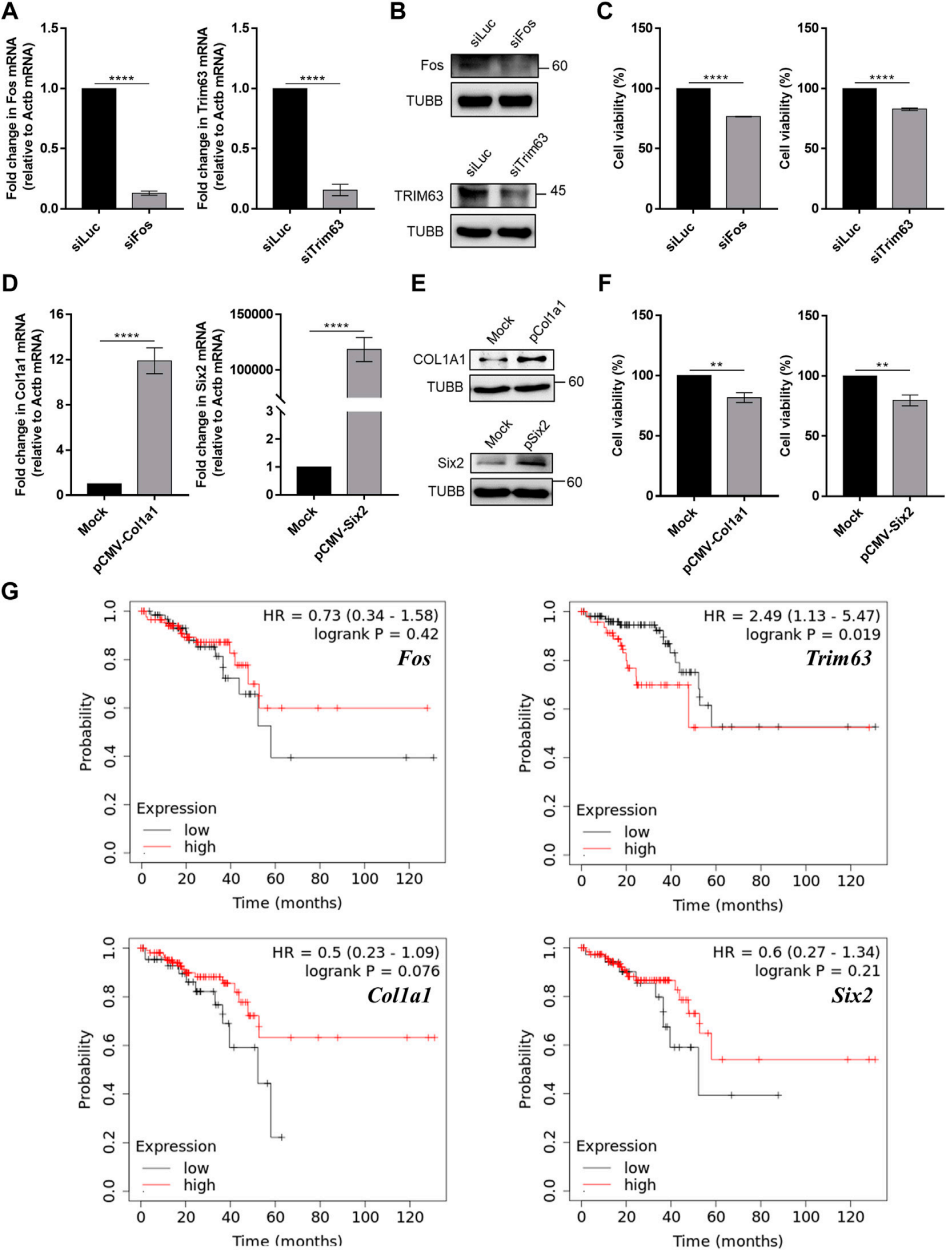
Modulation of skeletal muscle gene expression affects cancer cell viability *in vitro*. **(A)** Knockdown efficiency of siRNAs against *Fos* and *Trim63* in CT26 cells was confirmed using RT-qPCR. **(B)** The protein expression of Fos and Trim63 in siRNA-transfected cells. **(C)** Cell viability of CT26 cells with knockdown of skeletal muscle genes. **(D)** Overexpression of *Col1a1* and *Six2* resulted in increased mRNA expression, as confirmed using RT-qPCR. **(E)** Increased mRNA expression resulted in increased protein levels. **(F)** Viability of CT26 cells when *Col1a1* or *Six2* was overexpressed. The overexpression of *Col1a1* resulted in an about 20% reduction in CT26 cell viability. **(G)** Plots of Kaplan–Meier estimates for the overall survival rate of rectum adenocarcinoma patients based on the expression of skeletal muscle genes. With the exception of *Fos,* our anticancer candidate genes showed an expected association with the overall survival rate. For RT-qPCR and cell viability data, the average of three biological replicates is shown, with error bars indicating the standard error of the mean. For statistical analysis, the student’s t-test was performed against the control (siLuc or Mock), and the statistical significance was indicated with a number of *. **p-value < 0.01, ***p-value < 0.001, ****p-value < 0.0001.

We further examined the effects of modulating the expression of *Fos* and *Col1a1* genes in an *in vitro* cell line system. Using siRNAs, we knocked down the expression of *Fos* in CT26 cells and examined its effect on cell proliferation. We targeted *Fos* expression because our mRNA-seq analysis revealed that *Fos* expression was significantly decreased in mice that underwent high-intensity aerobic exercise, while its expression was increased in mice with cancer. Therefore, we investigated whether the downregulation of *Fos* expression might partly mimic the effect of exercise in terms of preventing cancer development. The results confirmed that siRNA transfection caused over 80% reduction in target mRNA expression and a remarkable decrease in protein levels (Figures 6A,B). Under these conditions, we examined cancer cell proliferation using the MTT assay. Downregulation of *Fos* reduced murine colorectal cancer cell proliferation by approximately 20% (Figure 6C, *p* = 1.8e-5).

In addition to *Fos,* we also investigated the effect of high-intensity aerobic exercise on *Col1a1* expression. *Col1a1* is related to the PI3K-AKT pathway and proteoglycans in cancer signaling pathways (Supplementary Data S1). For *Col1a1*, we performed an analogous experiment, but this time, we examined the effect of overexpression of *Col1a1* as its expression was increased in mice that underwent high-intensity aerobic exercise and decreased in cancer-bearing mice. We confirmed that the expression of a plasmid encoding *Col1a1* resulted in increased *Col1a1* mRNA and protein expression (Figures 6D,E). Moreover, the overexpression of *Col1a1* resulted in an approximately 20% reduction in CT26 cell viability (Figure 6F, *p* = 0.02).

Although they are not associated with immune-related signaling pathways, we also examined the expression of *Trim63* and *Six2*, based on the negative correlation observed in expression upon the induction of high-intensity aerobic exercise and cancer. Similar to *Fos*, *Trim63* showed decreased expression in the exercised mice, while it showed increased expression in the cancer-bearing mice. *Six2* also showed a negative correlation, but the opposite was observed in direction. We downregulated *Trim63* using siRNA and confirmed the knockdown efficiency using RT-qPCR and western blotting (Figures 6A,B). Similar to *Fos* knockdown, downregulation of *Trim63* also resulted in a significant reduction in CT26 cell viability (Figure 6C, *p* = 6.5^–4^). Similarly, we overexpressed *Six2* and confirmed the increased expression using RT-qPCR and western blotting (Figures 6D,E). Overexpression of *Six2* also resulted in an approximately 20% decrease in cell viability (Figure 6F, *p* = 0.02).

Finally, we analyzed the clinical significance of the four exercise-regulated genes for rectum adenocarcinomas using the Kaplan–Meier Plotter (Nagy et al., 2021). With the exception of *Fos*, we found that genes regulated by exercise were related to the overall survival of cancer patients. Patients with increased expression of *Trim63* showed an inferior overall survival rate (Figure 6G). Notably, *Col1a1* and *Six2* showed the opposite associations. We found that patients with low expression of *Col1a1* and *Six2* showed inferior overall survival rates (Figure 6G). These data were consistent with our CT26 cell viability analysis. Collectively, our analyses confirmed that the genes whose expression showed a negative correlation between exercise and cancer might be responsible for the cancer-preventive effect of high-intensity aerobic exercise.

## 4 Discussion

In this study, we used a mouse cancer model to investigate changes in the gene expression profiles upon high-intensity aerobic exercise and the molecular effects of exercise on cancer prevention. Consistent with the results of a previous study (Jee et al., 2016a), high-intensity aerobic exercise increased the survival rate, quality of life, and muscle hypertrophy in a mouse cancer model. We employed high-throughput sequencing to examine DEGs in high-intensity aerobic exercise and cancer-bearing mice. More importantly, we identified genes (e.g., *Trim63*, *Fos*, *Col1a1*, and *Six2*) that were regulated by high-intensity aerobic exercise, which adversely affected colorectal cancer development, and confirmed their effects *in vitro* using knockdown and overexpression systems.

### 4.1 Suppressive Effect of Skeletal Muscle-Derived Genes on Cancer Growth

We hypothesized that unknown skeletal muscle-derived factors play a role in suppressing cancer growth and metastasis. These factors could be regulated by physical activities, which may account for the preventive effect of exercise on carcinogenesis. Our previous study, using mouse cancer models, revealed the positive effects of high-intensity aerobic exercise rather than moderate-intensity aerobic exercise on various parameters, including a 100% survival rate (Jee et al., 2016a). The present study extended these observations by analyzing the genes regulated by high-intensity aerobic exercise. Through analysis of mRNA transcriptome, we identified 23,282 genes expressed in the gastrocnemius muscle, lungs, and heart. Of these genes, those with significantly altered expression were identified. Among them, we confirmed that 11 genes from the skeletal muscle (*Col3a1*, *Col5a2*, *Tnnc1*, *Col1a1*, *Osr2*, *Ifrd1*, *Trim63*, *Asb2*, *Maff*, *Myl6b*, and *Fos*), five from the lungs (*Cflar*, *Actn3*, *Myoz2*, *Il15*, and *Acta1*), and two from the heart (*Myoc*, and *Trim63*) showed reverse expression changes between high-intensity aerobic exercise and cancer-bearing. We focused on identifying epigenetically regulated genes in the skeletal muscle and selected a few candidate genes that might account for the cancer-preventive effects of aerobic exercise. These included *Fos*, *Trim63*, *Col1a1*, and *Six2*, two of which were downregulated by high-intensity aerobic exercise, while the other two were upregulated by high-intensity aerobic exercise. We also examined the effects of modulating their gene expression in cancer and observed significantly opposing effects on cancer cell viability by approximately 20%.

*Fos* and *Trim63* were selected for the knockdown experiments. Tripartite motif (Trim)-related families are renowned as muscle atrophying effector genes (Yang et al., 2014). *Trim24* has been identified as an oncogene in colorectal cancer using lentivirus-mediated RNA interference knockdown in HCT116 human colorectal cancer cells, which significantly decreased cell proliferation (Cohen et al., 2012; Wang J. et al., 2014). *Trim63*, also known as *Murf1*, maintains muscle protein homeostasis by acting on sarcomere-related proteins, such as microtubules and myosin heavy chain (McElhinny et al., 2002; Chen et al., 2012). It is involved in the atrophy of skeletal muscles and myocardium (Tan et al., 2017). Trim63 protein localizes to the Z-line and M-line lattice of myofibrils and interacts with numerous signaling pathways, such as the microtubule-dependent signaling pathway in muscle or cancer regulating SUMO-related pathways (Centner et al., 2001; Hu and Jiang, 2019). It is also suppressed by the Wnt/β-catenin signaling pathway in breast cancer and affects cell proliferation and migration (Bowen et al., 2017).

In addition to *Trim63,* our results on *Fos* are also consistent with previous reports, where the inhibition of *Fos* suppressed colon carcinoma tumor growth in athymic mice as well as the progression of ovarian and breast cancer (Pandey et al., 2012; Oliveira-Ferrer et al., 2014; Li et al., 2019). Bioinformatic studies have also shown that, regardless of the tumor type, *Col1a1* acts as a cancer suppressor (Lu et al., 2005). Lastly, the transcription factor *Six2*, which is involved in kidney development, controls E-cadherin and promotes stemness in the lung and breast cancer cells (Li et al., 2020; Weber et al., 2008; Wang C.-A. et al., 2014).

### 4.2 Relationship Between the Skeletal Muscle-Derived Factors Identified in This Study and Cancer Suppression

In the current study, along with a significant impact on hormone and protein secretion, exercise resulted in a more remarkable effect on the regulation of the skeletal muscle than that of the lungs or heart. In particular, the expression of secretion-related genes (e.g., *BNDF, BRSK1*, and *Ccnd1*) in the gastrocnemius muscle during high-intensity aerobic exercise suggests their potential role in cancer suppression. This is consistent with previous studies that reported the importance of muscle-derived secretion factors and their effects on other tissues throughout the body (Hojman et al., 2011; Hamrick, 2012; Hou et al., 2019). Our study also revealed differential expression of growth factor-related genes (*Fibroblast growth factor 2/6/10/18, Fibroblast growth factor receptor 2, Epidermal growth factor-containing fibulin-like extracellular matrix protein 2, Insulin-like growth factor 1, Insulin-like growth factor binding protein 5,* and *Platelet-derived growth factor*), interleukin-related gene (IL15), and other myokine or cytokine-related genes (*interferon-related developmental regulator1*, and *Myostatin*). In the future, it will be interesting to investigate how high-intensity aerobic exercise affects the body through the secretion of these factors.

In addition to the four genes (*Fos*, *Col1a1*, *Trim63*, and *Six2*), our study identified other potential molecular factors of high-intensity aerobic exercise, which may prevent cancer proliferation. A key factor is *IL15*. Studies on the effects of myokines, such as interferons and interleukins, on cancer reported that the muscle-derived gene *IL15* triggers the immune system by forming the IL15+IL15R complex. It then stimulates the spleen cells and activates the natural killer (NK) T, CD8^+^ T, and CD4^+^ T cells (Carson and Caligiuri, 1998; Pedersen and Hojman, 2012; Idorn and Hojman, 2016). Moreover, the IL15+IL15R complex increases the number of effectors and central memory cells, which are anticancer immune cells involved in inducing metabolic differentiation of T lymphocyte-related subsets in the spleen (Yang et al., 2011; Klebanoff et al., 2016; Thi et al., 2019). Although IL15 alone is not involved in any major cancer signaling pathways, the IL15+IL15R complex can suppress CT26 colon cancer cells through NK cells (Thi et al., 2019), which is consistent with the findings on the upregulation of *IL15* by intensive aerobic exercise in the current study.

### 4.3 Effect of Exercise on Improving Body Wellness in Cancer Patients, and the Type of Exercise That Can Effectively Suppress Cancer

In an epidemiologic study with 500,000 participants, those who engaged in moderate-intensity exercise three to four times a week (men) and once or twice a week (women) showed a significantly decreased incidence rate of colon cancer (by 54% and 47%, respectively) (Jee and Kim, 2019). The findings of this epidemiological study on human subjects suggest that colon cancer-targeted effects vary depending on sex and the amount of exercise. Our previous (Jee et al., 2016a) and current studies also showed the cancer-suppressive effect of high-intensity aerobic exercise. Other studies demonstrated that resistant exercise on the animal cancer model mitigates tumor growth, tumor grade, viable tumor area, tumor cell proliferation, and myofiber atrophy-causing cancer cachexia *via* attenuating some key markers such as TNF-α, IL-6, Atrogin1, and oxidative damage (Padilha et al., 2019; Padilha et al., 2021). In addition, we also found an effect of exercise on cancer cachexia. Consistent with the findings of previous studies (Deuster et al., 1985; Penna et al., 2011), cancer resulted in decreased food intake and body weight, two indicators of cancer cachexia. Notably, mice subjected to high-intensity aerobic exercise showed no significant changes in food intake and body weight when compared to healthy control mice. A previous study using lung cancer-bearing mice showed the importance of changes in the thermogenic gene (*Dios2*) and skeletal muscle atrophy-related genes (*Atrogin1* and *Murf1*) in driving cancer cachexia (Kir et al., 2014). The cancer cachexia increases the resting energy expenditure by browning fat thermogenesis. The thermogenic gene *Dios2* and two muscle atrophy-related genes, *Atrogin1* and *Murf1,* were upregulated by parathyroid hormone-related protein (PTHrP), which is responsible for most of the cancer cell-derived browning adipose cell activity. This study argued that neutralizing the PTHrP could block adipose tissue browning and subsequent loss of muscle mass and strength. Interestingly, our mRNA-seq data also showed changes in the expression of *Dios2* in the lungs and *Atrogin1* and *Murf1* in the skeletal muscles of cancer-bearing mice, and the above mechanism may apply. Notably, aerobic exercise rescued the cancer-driven changes in *Astogin1* and *Murf1* expressions. Accordingly, our analysis may justify the reduced muscular strength and exercise capacity during cancer cachexia and how aerobic exercise can ameliorate cancer cachexia.

The tumor-suppressive effects of exercise also vary according to the exercise type, intensity, frequency, and type of cancer. Therefore, creative exercise interventions for individual patients should be developed. Different types of exercise, such as aerobic resistance training, elicit disputable effects, and the optimal intensity and frequency are important factors that determine the potential benefits. In this study, mice were subjected to high-intensity aerobic exercise (90% of the maximal heart rate) as a model to study the importance of preventive exercise and an exercise-centered lifestyle. However, adequate mouse models mimicking human subjects, who do not exercise regularly, should be developed to study the differences and compare the signaling pathways between the two groups to emphasize the exercise-centered lifestyle to pursue a healthy state of the body.

The mouse cancer model used in this study was established after its physical fitness was raised to imitate the effects of regular physical activity. In the future, it will be important to revalidate the effects of high-intensity aerobic exercise in human subjects. Pollán et al. (2020) indicated that the most effective behavioral interventions that may bring health improvements and changes in the lifestyles of patients vary depending on the intensity and duration of the physical activity. However, the type of workout effective for each type of cancer is unclear and remains to be investigated.

Exercise seems to prevent cancer primarily by reducing the circulating levels of hormones and growth factors, such as insulin-like growth factor 1. Preclinical studies have shown that exercise induces hyperphosphorylation of the retinoblastoma protein, which causes the phosphorylation of β-catenin and subsequent reduction in the expression of multiple microRNAs involved in a wide range of biological functions, including cellular proliferation, differentiation, regulation of inflammation, antioxidation, glucose transportation, angiogenesis, and muscle contraction (Baltgalvis et al., 2008; Ju et al., 2008; Zhu et al., 2008; Jiang et al., 2009; Horak et al., 2018). Various reports have described the potential mechanisms underlying the anticancer effects of exercise. Most of these reports have focused on the suppression of cancer with an enhanced immune system, such as the stimulation of natural killer cells. In contrast, the present study focused primarily on the effects of exercise on the skeletal muscle, suggesting a role of myokines in this tissue. Although not much is known about their role in this context, myokines are considered to have a direct anticancer effect *via* the secretion of acidic proteins (Horak et al., 2018). In our results, *IL15* and *Myostatin* were listed in the DEGs of the skeletal muscle.

The practical use of the results from the current study requires future investigation of aerobic exercise using human subjects. In particular, the expression of the anticancer candidate genes identified in this study needs to be examined using human skeletal muscle under the same condition. In addition, the human analogous of aerobic exercise that induces sufficient changes in anticancer gene expression needs to be devised. Lastly, we anticipate that there will be difficulty in making cancer patients to undergo high-intensity aerobic exercise.

Analyzing the effect of exercise on the liver in the cancer model was important because the liver tissue is closely associated with maintaining body weight, insulin sensitivity, and chronic inflammation. Moreover, we also found that central cancer regulating pathways related to the risk of hepatocellular carcinoma (Saran et al., 2018), such as the AMPK pathways, were affected by high-intensity aerobic exercise. Another limitation of the study includes the lack of *in vivo* validation of candidate genes through CRISPR knockout mice. Particularly, *in vitro* transfection of *Six2* resulted in too high expression of the target gene. *In vivo* validation using a more physiologically relevant promoter will further support our results. These are the limitations of the current study, which warrant future analysis. In particular, the transcriptome-wide gene expression changes in the livers of cancer-bearing and high-intensity aerobic exercise mice may provide important insights into the effects of exercise on cancer prevention and cancer cachexia. Despite these limitations, our study clearly suggests that the high-intensity aerobic exercise resulted in changes in the expression of muscle-derived anticancer genes (*Fos*, *Col1a1*, *Trim63*, *and Six2*) that may account for the cancer-preventive effect of aerobic exercise.

In conclusion, our study confirmed the effects of high-intensity aerobic exercise in a mouse model of cancer and revealed aerobic exercise-derived gene expression changes in the skeletal muscle, lungs, and heart. Selected genes derived from the skeletal muscle of the high-intensity aerobic exercise group displayed an approximately 20% decrease in cancer cell viability when properly modulated. Further studies on the impact of exercise on cancer, for example, different types of physical activities and analyses conducted prior to and following an exercise regimen, are warranted.

## Data Availability

The datasets presented in this study can be found in online repositories. The names of the repository/repositories and accession number(s) can be found below: GEO, accession number(s) GSE191281, GSE191283, GSE191284.

## Abbreviations

Asb, ankyrin repeat and SOCs box containing; Col1a1, collagen type 1 alpha 1 chain; CT26, colon tumor 26; ILs, interleukins; mRNA seq, messenger ribonucleic acid sequencing; Maff, MAF BZIP transcription factor F; Osr, odd-skipped related transcription factor; pcDNA, plasmid cloning deoxyribonucleic acid; siRNA, small interfering RNA; Trim63, tripartite motif-containing 63; Tnnc, troponin c; Ifrd, interferon-related developmental regulator.
