# Supplementary material for: High-Intensity Aerobic Exercise Suppresses Cancer Growth by Regulating Skeletal Muscle-Derived Oncogenes and Tumor Suppressors
Source: Front Mol Biosci. 2022 Jun 21;9:818470. doi: 10.3389/fmolb.2022.818470 (PMC9254717; doi:10.3389/fmolb.2022.818470)

Supplementary Table ST1. siRNA sequence

Gene	Sense(5'-3')	Antisense (5'-3')
siLuc	CUUACGCUGAGUACUUCGA	UCGAAGUACUCAGCGUAAG
siFos	UAGUAGAGCAUGUGAGUCAUU	UGACUCACAUGCUCUACUAUU
siTrim63	GGACUGAAUUUGUGUUAUAUU	UAUAACACAAAUUCAGUCCUU

Supplementary Table ST2. Primer sequences for RT-qPCR

Gene	Forward primer (5′-3′)	Reverse prime (5′-3′)
GAPDH	AAGAGGGATGCTGCCCTTAC	ATCCGTTCACACCGACCTTC
Col3a1	GTCCAGGGATACGGGGTATG	CAGGGAAACCCATGACACCA
Col5a2	TGGGGACTGATGGTACACCT	GGATCACCCGATTGTCCTCG
Myl6b	TGGGGAACCCCAAAAACGAG	ACGAAGCCCCTCTAGGTAGT
Col1a1	GAGAGGTGAACAAGGTCCCG	AAACCTCTCTCGCCTCTTGC
Ifrd1	GCTTCACATCAGCTCGCTTC	TCGCCAGCAGCAATTCTCAT
Fos	CCTCCTGACACGGTCTTCAC	GATCTGTCTCCGCTTGGAGT
Asb2	AGTCTGTCTCCCGCAATGAC	GTGTTGATGTCTGCACCATGC
Tnnc1	TTGACAAAAACGCTGATGGCT	CCGTGCAAGACCAGCATCTA
Osr2	CCTTCCAGCCCTACACAAGG	GCTTTCTGTCCGGGTTCAGT
Maff	ATCACCATCGTCAAGTCGG	GTCACTACAACGAGTGGCAGA
Six2	TTCAAGGAGAAGAGCCGCAG	TTCTCCCTTTCCTTGGCCTC
Trim63	CCAGGCTGCGAATCCCTAC	GTTTTCCACCAGCAGGTTCC
Il15	ACTGAGGCTGGCATTCATGT	CTGTCAGTGTATAAAGTGGTGTCA
Cflar	GGCAGAGGCAAGATAGCCAA	ATCTTGCTCCTTGGCTGGAC
Cacna1s	AAGCCCAGGGGCTATTTTGG	TCCACCCAGGCAATACAGTC
Actn3	GAGCTCGACTACCATGAGGC	GCCAGTTATTGAAGGGGGCT
Myoz2	ACAGGGTTGCCACTCCATTT	TAAAGCATCGTCTGCCCGAA
Acta1	GACCACAGCTGAACGTGAGA	GAAACGCTCATTGCCGATGG
Ar	CCAGATGGCGGTCATTCAGT	ACTTGTGCATGCGGTACTCA
Myoc	ACGACACTAAAACGGGGACC	TTCTGGCCTTTGCTGGTAGG

Supplementary Data 1. Functional annotation of key DEGs. The functional annotation analysis of selected DEGs reveals an association of the major cancer signaling pathways.

Cflar	CASP8 and FADD-like apoptosis regulator(Cflar)	Related Genes	Mus musculus
KEGG_PATHWAY	NF-kappa B signaling pathway, Apoptosis, TNF signaling pathway, Chagas disease (American trypanosomiasis),		
Fos	FBJ osteosarcoma oncogene(Fos)	Related Genes	Mus musculus
KEGG_PATHWAY	MAPK signaling pathway, cAMP signaling pathway, Osteoclast differentiation, Toll-like receptor signaling pathway, T cell receptor signaling pathway, B cell receptor signaling pathway, TNF signaling pathway, Circadian entrainment, Cholinergic synapse, Dopaminergic synapse, Estrogen signaling pathway, Prolactin signaling pathway, Oxytocin signaling pathway, Amphetamine addiction, Salmonella infection, Pertussis, Leishmaniasis, Chagas disease (American trypanosomiasis), Hepatitis B, HTLV-I infection, Herpes simplex infection, Pathways in cancer, Colorectal cancer, Choline metabolism in cancer, Rheumatoid arthritis,		
Actn3	actinin alpha 3(Actn3)	Related Genes	Mus musculus
KEGG_PATHWAY	Focal adhesion, Adherens junction, Tight junction, Leukocyte transendothelial migration, Regulation of actin cytoskeleton, Amoebiasis, Viral carcinogenesis, Systemic lupus erythematosus,		
Ar	androgen receptor(Ar)	Related Genes	Mus musculus
KEGG_PATHWAY	Oocyte meiosis, Pathways in cancer, Prostate cancer,		
Cacna1s	calcium channel, voltage-dependent, L type, alpha 1S subunit(Cacna1s)	Related Genes	Mus musculus
KEGG_PATHWAY	MAPK signaling pathway, Calcium signaling pathway, cGMP-PKG signaling pathway, cAMP signaling pathway, Cardiac muscle contraction, Adrenergic signaling in cardiomyocytes, Vascular smooth muscle contraction, Retrograde endocannabinoid signaling, Cholinergic synapse, Serotonergic synapse, GABAergic synapse, Insulin secretion, GnRH signaling pathway, Oxytocin signaling pathway, Renin secretion, Aldosterone synthesis and secretion, Alzheimer's disease, Hypertrophic cardiomyopathy (HCM), Arrhythmogenic right ventricular cardiomyopathy (ARVC), Dilated cardiomyopathy,		
Col1a1	collagen, type I, alpha 1(Col1a1)	Related Genes	Mus musculus
KEGG_PATHWAY	PI3K-Akt signaling pathway, Focal adhesion, ECM-receptor interaction, Platelet activation, Protein digestion and absorption, Amoebiasis, Proteoglycans in cancer,		
Col3a1	collagen, type III, alpha 1(Col3a1)	Related Genes	Mus musculus
KEGG_PATHWAY	PI3K-Akt signaling pathway, Focal adhesion, ECM-receptor interaction, Platelet activation, Protein digestion and absorption, Amoebiasis,		
Col5a2	collagen, type V, alpha 2(Col5a2)	Related Genes	Mus musculus
KEGG_PATHWAY	PI3K-Akt signaling pathway, Focal adhesion, ECM-receptor interaction, Platelet activation, Protein digestion and absorption, Amoebiasis,		
Il15	interleukin 15(Il15)	Related Genes	Mus musculus
KEGG_PATHWAY	Cytokine-cytokine receptor interaction, Jak-STAT signaling pathway, TNF signaling pathway, Intestinal immune network for IgA production, HTLV-I infection, Herpes simplex infection, Rheumatoid arthritis,		
Tnnc1	troponin C, cardiac/slow skeletal(Tnnc1)	Related Genes	Mus musculus
KEGG_PATHWAY	Calcium signaling pathway, Cardiac muscle contraction, Adrenergic signaling in cardiomyocytes, Hypertrophic cardiomyopathy (HCM), Dilated cardiomyopathy,		

Supplementary Data 2. The KEGG pathway analysis of DEGs. The KEGG molecular annotation was used to analyze the inter-network map of the top 285 differentially expressed skeletal muscle-related genes from the mRNA-seq analysis of gastrocnemius muscle (http://www.kegg.jp/kegg/mapper.html). Pathways in cancer is shown. The colored boxes represent genes present in our study. Red and blue indicate up- and downregulated by exercise, respectively (comparisong E(+)T(-) and E(-)T(-)). Renowned cancer pathways (e.g., MAPK, p53, etc) are evident.

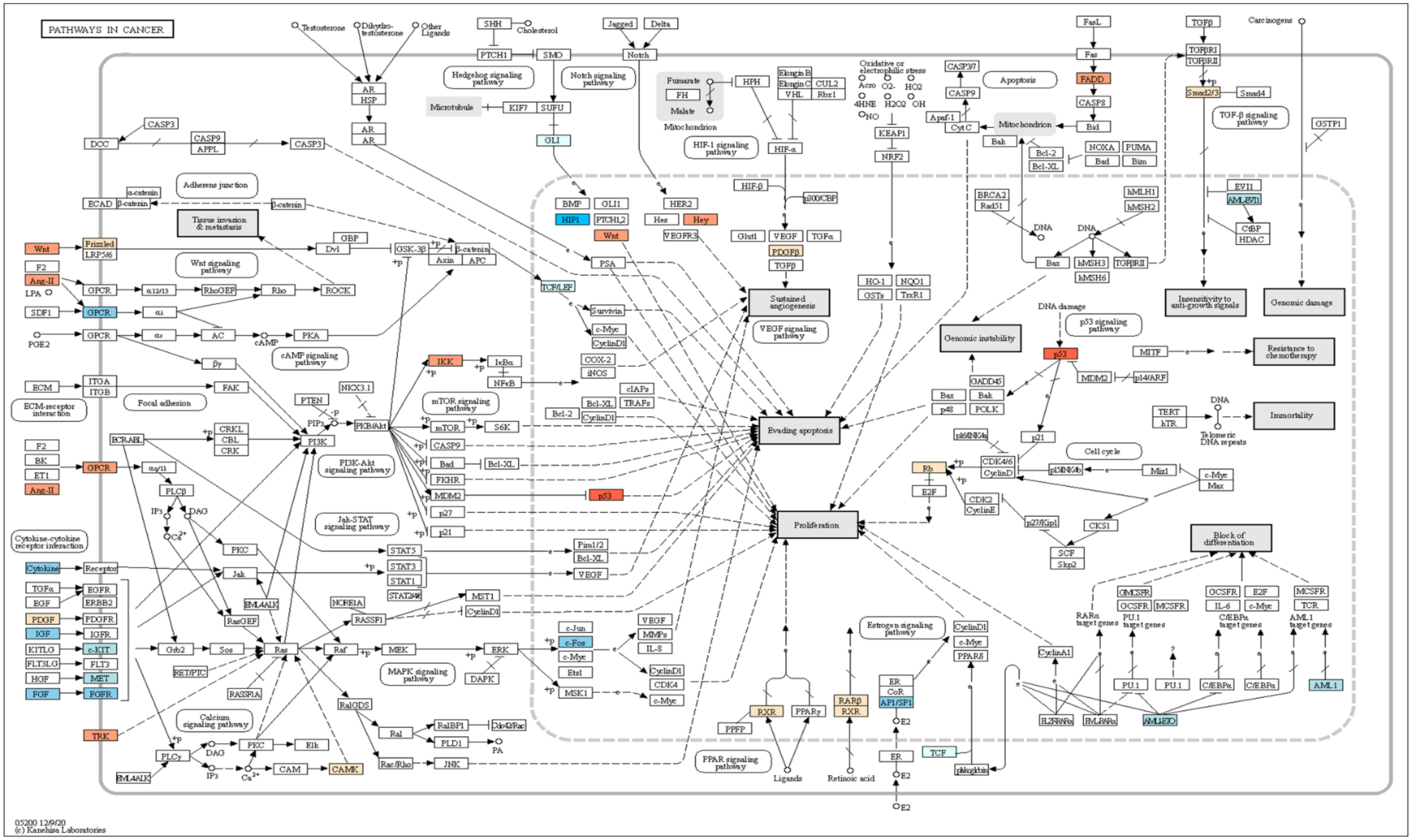

Supplementary Data 3. Protein-protein interaction of selected DEGs. The molecular interaction of the 40 selected differentially expressed skeletal-muscle related genes at the protein level was analyzed using the String-database tool (http://string-db.org). These genes showed at least two fold difference in expression with normalized read count of at least four in all three-way comparisons (E(+)T(-) vs. E(-)T(-), E(-)T(+) vs. E(-)T(-), and E(+)T(+) vs E(-)T(+)), which includes the final four genes used in our in vitro experiment (*Fos, Trim63, Col1a1,* and *Six2*).

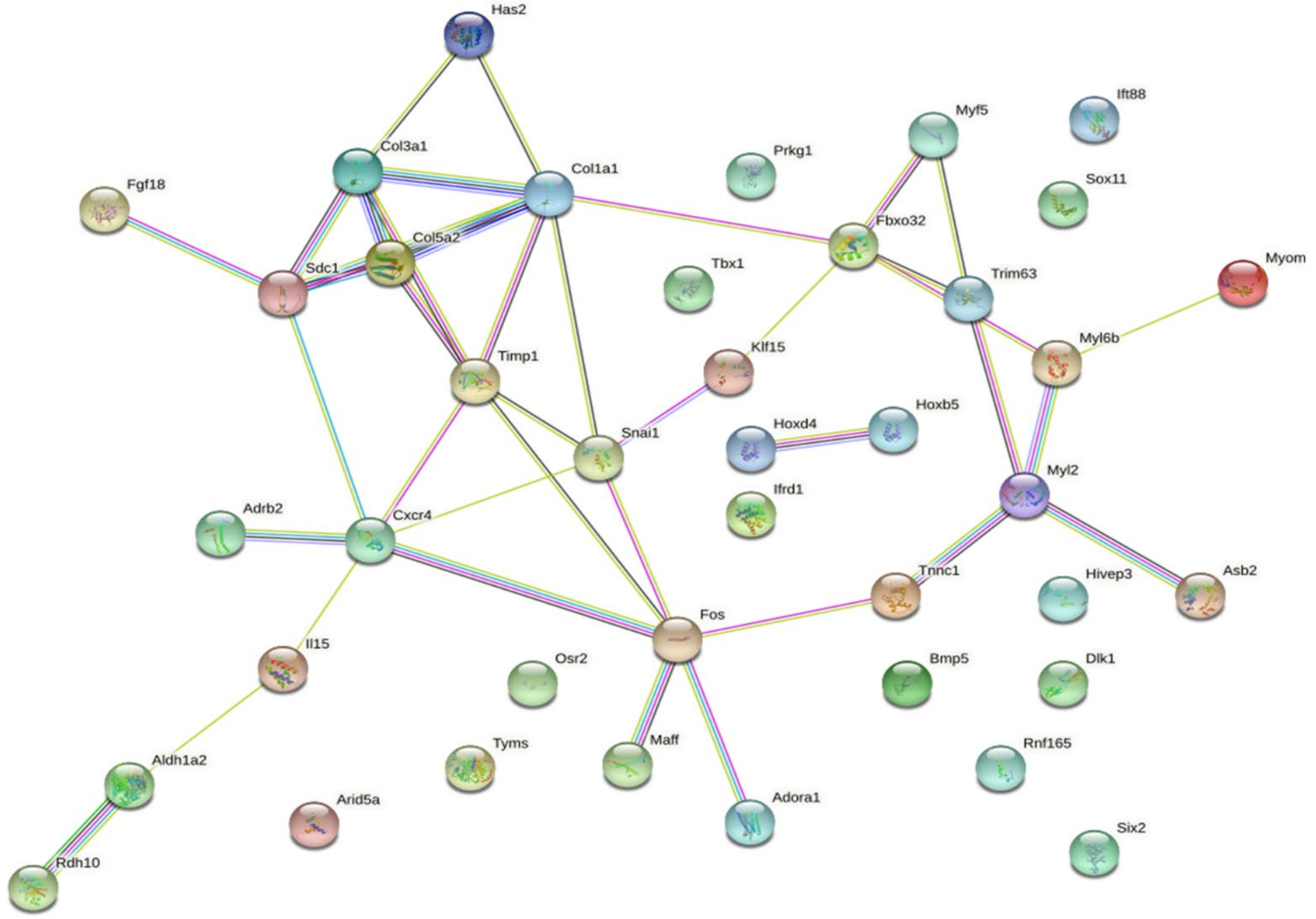

Supplement: Supplementary file 1 [file DataSheet1.PDF]
